# A Facile Method to Prepare Silver Doped Graphene Combined with Polyaniline for High Performances of Filter Paper Based Flexible Electrode

**DOI:** 10.3390/nano9101434

**Published:** 2019-10-10

**Authors:** Shasha Jiao, Tiehu Li, Chuanyin Xiong, Chen Tang, Hao Li, Tingkai Zhao, Alei Dang

**Affiliations:** 1 School of Materials Science and Engineering, Northwestern Polytechnical University, Xi’an 710072, China; litiehu@nwpu.edu.cn (T.L.); tangchen@mail.nwpu.edu.cn (C.T.); lihao@nwpu.edu.cn (H.L.); ztk@nwpu.edu.cn (T.Z.); dangalei@nwpu.edu.cn (A.D.); 2 School of Bioresources Chemical and Materials Engineering, Shaanxi University of Science and Technology, Xi’an 710072, China; xiongchuanyin@sust.edu.cn

**Keywords:** supercapacitor, grapheme, silver, polyaniline, filter paper

## Abstract

A flexible filter paper based composite electrode was prepared via the convenient one-step synthesis of silver doped graphene for the first time, followed by in-situ polymerization of aniline monomers. Using L-ascorbic acid for simultaneous reduction of grapheme oxide and silver nitrate, we provided a new and green method to prepare graphene hybrid sheets without toxicity. It was found that the as-fabricated hybrid electrode formed a three-dimensional porous architecture, which not only increased the specific surface area of composite, but also facilitated the ion diffusion of the electrolyte. In addition, according to the tests of electrochemical performances, the flexible hybrid electrode subsequently exhibited exceptional specific capacitance of 437.3 F/g, energy density of 1133.5 W·h/kg and power density of 88.8 kW/kg, respectively. Meanwhile, the as-prepared hybrid demonstrated a good cycling stability with only 10.99% specific capacitance deterioration after 5000 times of cycling. This preparation technology presented here shows great potential for the development and application of wearable and portable energy storage devices, particularly for flexible supercapacitors. Moreover, this study puts forward a general, simple and low-cost route of fabricating a novel flexible electrode on a large scale, eventually for environmental protection.

## 1. Introduction

With the aggravation of environmental pollution and the over exploitation of natural resources, the development and utilization of renewable energy has aroused extensive attention and research throughout the world. Among numerous energy storage systems, the supercapacitor is the most promising device by virtue of its excellent properties [1,2,3,4]. Generally, depending on the charge storage mechanisms, supercapacitors can be divided into two general categories: Electric double layer capacitors (EDLCs) and faradaic pseudocapacitors [5,6]. For EDLCs, capacitance relies on the charge accumulation of the ions absorbed from the electrolyte through electrostatic force. Carbon nanomaterials (e.g., carbon nanotubes and graphene) with large surface area and high activity are the most popular materials for EDLC electrodes in recent years [7]. However, pseudocapacitance depends on the reversible redox reactions taking place on the surface of the electrode. There are two types of materials that can be used for pseudocapacitors. These are transition metal oxides (e.g., manganese dioxide) and conductive polymers (e.g., polyaniline), respectively [8]. In order to combine the advantages of EDLCs and pseudocapacitors, hybrid supercapacitors were fabricated by using the carbon nanomaterial/metal oxide/ polyaniline composite as electrodes [9,10,11]. Owing to the development of portable and wearable technology, electronic devices with good flexibility and low cost are increasingly being sought after because of the excellent electrochemical performances upon mechanical bending [12,13,14,15]. Recent years have witnessed the great efforts of researchers to develop and find the low-cost and simple technology of preparing flexible, lightweight and ultrathin substrates, which is considered as one of the crucial steps in preparing flexible supercapacitors with high performances [16,17].

Among the numerous flexible substrates, paper, with its significant advantages of low cost, good flexibility as well as environmental-friendly properties; enables some novel and interesting applications, such as wearable devices. Generally, paper-based supercapacitors rely primarily on the hybrid of conductive and active materials with cellulose fibers as matrix, forming a crosslinked network structure, which could improve the strength, toughness and other properties. Combined with electrochemical activities (graphene, carbon nanotubes, polyaniline and polypyrole), the modified cellulose fibers of paper are beneficial to the surface area, and contact between electrode, electrolyte and large pore size is in favor of electrolyte transport.

Due to the spatial interconnected and porous structure, dropped filter paper (FP), one of the recyclable papers generated by daily chemical experiments, is becoming a more and more competitive candidate for flexible supercapacitor-making at present [18,19]. Similar to other papers, the filter paper mainly composes of cellulose fibers with a three-dimensional hierarchical architecture. Particularly, in comparison with other common flexible substrates, filter paper has large surface roughness, better absorbability and porousness, which shows great superiority for some applications, such as for many energy devices in which large surface roughness is in need. People can also utilize the porosity of FP to prepare a cellulose fiber-based composite by soaking with chemical active materials.

However, like other paper-based supercapacitors, the low electrochemical activity and poor electrical conductivity of FP seriously hindered the development and application in portable and wearable electric devices. To overcome this drawback, it is of great significance to explore new modification methods of paper-based substrates. The most frequently used way is to deposit various carbon nanomaterials on the surfaces of cellulose paper, which can improve electrochemical performances remarkably [20]. Another straightforward approach is to prepare metallic nanoparticles to increase the conductivity of paper-based substrates. Graphene, a two-dimensional honeycomb-shaped sheet of carbon atoms, has attracted great research interest in material science because of its excellent chemical–physical, optical, electrical and other properties, which make it widely used in supercapacitors, display screens, solar cells, batteries and electrical circuits [21,22,23,24]. Polyaniline (PANI) is one of the typical conducting polymers with high conductivity, superior electrical redox properties, high specific capacitance and good stability [25,26]. Combining the excellent properties of PANI and graphene, high performance graphene/PANI composites have gained considerable attention in preparing flexible hybrid supercapacitors under effective, economic and environmentally friendly conditions [27,28,29]. Cong et al. prepared a paper-like graphene-PANI composite with high flexibility by a facile method. The results showed excellent electrochemical performances with an enhanced specific capacitance of 763 F/g and 82% capacity retention after 1000 cycles [30]. In the present scenario, different types of nanometallic materials have been employed to prepare flexible nanocomposites for supercapacitors because of its unique structure and extraordinary properties. Zhang et al. successfully fabricated a flexible Ni/MnO_2_-filter paper electrode with filter paper as a substrate. The areal specific capacitance of the as-prepared electrode reaches to 1900 mF/cm^2^ at a scanning rate of 5 mV/s [18]. Among the common metal materials, silver nanoparticles have been frequently investigated because of their high conductivity, simple preparation and good environmental stabilities. Zhi et al. coated the ordered mesoporous carbon on the surface of graphene foam, followed by the decoration with silver nanowires on the composite. The unique structure of silver nanowires/3D-graphene foam/ordered mesoporous carbon (Ag-GFOMC) exhibited an excellent specific capacitance of 213 F/g, energy density of 4.5 W h/kg and power density of 5040 W/kg, respectively [31].

Herein, in this paper, a three-dimensional flexible PANI/rGO/Ag/FP ultrathin electrode was reported using a facile method. To start with, silver nanoparticles and the reduced graphene oxide were grafted to the filter paper by a chemical reduction with L-ascorbic acid. Then polyaniline nanostructures were prepared via situ polymerization at room temperature. X-ray diffraction, Raman spectrum, scanning electron microscope and high-resolution transmission electronic microscope were jointly employed to confirm the structure and morphology of PANI/rGO/Ag/FP hybrid, respectively. The electrochemical results showed an excellent specific capacitance of 437.3 F/g, energy density of 1133.5 W h/kg and power density of 88.8 kW/kg, respectively. The reason for this was that the silver nanoparticles, graphene and polyaniline combined with the porous filter paper produced synergistic effects in the hybrid. Besides, the specific capacitance retention of flexible electrode was up to 89.01% after 5000 cycles, indicating good cycling stability.

## 2. Materials and Methods

### 2.1. Materials

Graphite powder was procured by Risheng Graphite Ltd., Qingdao, China and the purity was greater than 99%. Filter paper (FP), 36% concentrated hydrochloric acid (HCl), 98% concentrated sulfuric acid (H_2_SO_4_), hydrogen peroxide (30%, H_2_O_2_), potassium permanganate (KMnO_4_), sodium nitrate (NaNO_3_), silver nitrate (AgNO_3_) and L-ascorbic acid (L-AA) were all purchased from Sanpu Fine Chemical Factory, Xi’an, China. All reagents were used as received.

### 2.2. Preparation of Graphene Oxide

Prior to the synthesis of electrochemical active substances on FP substrate, graphene oxide (GO) was prepared via the modified Hummers method by using natural graphite as raw material [32,33,34]. Then the obtained GO sheet was ground into a fine powder to make up of 0.2 mg/mL GO solution. Under the presence of ultrasound for 6 h, a uniform and well dispersed solution of GO was prepared.

### 2.3. Preparation of Flexible GO/AgNO_3_/FP Composite Film

**Figure 1:**
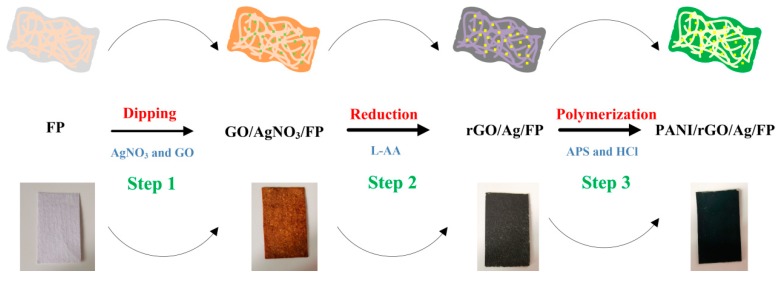
Fabrication of the flexible polyaniline (PANI)/rGO/Ag/filter paper (FP) hybrid electrode.

The first step in preparing GO/AgNO_3_/FP hybrid was to cut FP into a piece of 3 cm × 1 cm in size and mark it to serve as flexible substrate. Afterwards, the pre-cut samples were immersed in 0.1 mol/L AgNO_3_ solution prepared beforehand for 20 min and dried at room temperature. Then the FP cuttings impregnated with AgNO_3_ were put into 0.2 mg/mL GO suspension to adhere GO to the surface. After that, they were dried under vacuum. The procedures above with the alternative utilization of AgNO_3_ and GO mixture were repeated, respectively. The GO/AgNO_3_/FP hybrids were synthesized successfully after 20 cycles. Step 1 of Figure 1 provides an illustration to understand the procedures of preparation of flexible GO/AgNO_3_/FP composite film.

### 2.4. Preparation of Flexible rGO/Ag/FP Composite Film

The reduction reaction was carried out by L-AA at room temperature. First of all, the GO/AgNO_3_/FP samples were treated with 0.1 mg/mL L-AA solution until the surface turned brownish black. After several times of vacuum drying and water washing, the as-prepared reduced GO (rGO)/Ag/FP composite presented a silky-black color with uniformly distributed and ultrafine silvery highlights. The preparation process can be seen in Step 2 of Figure 1.

### 2.5. Preparation of Flexible PANI/rGO/Ag/FP Composite Film

Firstly, the as-prepared rGO/Ag/FP films were immersed in 0.2 M aniline and 1 M hydrochloride mixed aqueous solution for a while before polymerization. Then the standard oxidant of ammonium persulfate (0.25 M) in aqueous medium was poured into the above mixture to prepare PANI. Finally, after washing and drying for several times, PANI was successfully synthesized on the surface of rGO/Ag/FP composite. Figure 1 shows a schematic illustration of fabricating the flexible PANI/rGO/Ag/FP hybrid film.

### 2.6. Characterization

The chemical compositions of the samples were characterized by X-ray diffraction (XRD, D8-advance, Bruker, Cu-Kα, λ = 1.5418 Å, Bremen, Germany) and Raman spectroscope (Jobin Yvon LabRam HR800, France), respectively. Besides, the scanning electron microscopy (SEM, Hitachi S-5200, Tokyo, Japan) and high-resolution transmission electron microscopy (TEM, Hitachi H-7650, Tokyo, Japan) were employed to investigate the morphological properties and microstructures of the samples. The electrochemical performances of the as-synthesized electrode hybrids were characterized by an electrochemical workstation (CHI660 C, ChenHua Co., Ltd, Shanghai, China). The cyclic voltammetry (CV) measurements were performed under the potential window of −0.2~1 V with different scanning rates of 5, 10, 50 and 100 mV/s, respectively. The galvanostatic charge-discharge (GCD) tests were carried out under the current density of 1 A/g, 2 A/g and 5 A/g, respectively. The electrochemical impedance spectroscopy (EIS) technique was also used to study the reaction kinetics of PANI/rGO/Ag/FP composite material in the frequency range from 10 mHz to 100 kHz.

The electrochemical tests were carried out in 1 M Li_2_SO_4_ aqueous solution with a two-electrode system at room temperature. The as-prepared samples were directly used as the working electrode and counter electrode, respectively. Meanwhile, the specific capacitance can be obtained from the CV curves according to the calculation formula, below [35]:

(1) $$C_{m}=\int_{V_{1}}^{V_{2}}\mathrm{IdV}/2m\nu \Delta V$$

where C_m_ is the specific capacitance, I is the galvanostatic discharge current, m is the total mass of the active material in two electrodes, ν is the scanning rate during CV measurement and ΔV is the voltage range, respectively.

Moreover, the energy densities and power densities were calculated by the equations as follows:

(2) $$E=C_{m}\Delta V^{2}/2$$

(3)  P=E/Δt 

where E is the energy density, P is the power density and Δt is the discharge time during the charge-discharge measurements, respectively.

## 3. Results and Discussion

### 3.1. Structure and Morphology

**Figure 2:**
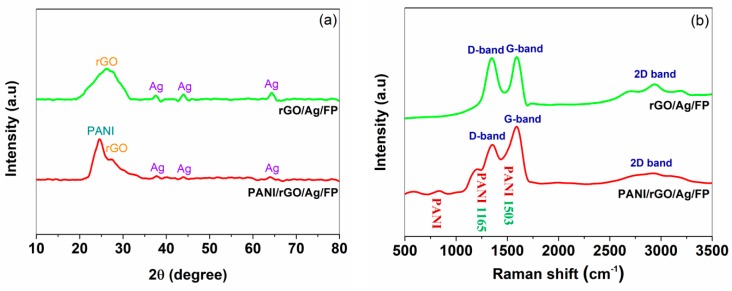
(**a**) XRD patterns of rGO/Ag/FP and PANI/rGO/Ag/FP electrodes, respectively. (**b**) Raman spectrum of rGO/Ag/FP and PANI/rGO/Ag/FP electrodes, respectively.

The structure of the as-prepared hybrids was observed and investigated by means of XRD, SEM and TEM. Figure 2a shows the XRD patterns of rGO/Ag/FP and PANI/rGO/Ag/FP composites, respectively. Apparently, the results indicated that the prepared rGO/Ag/FP showed a broad intense peak at around 26.6°, which was corresponding with the hexagonal structure of graphene. In addition, three weak diffraction peaks were also observed at 38.1°, 44.5° and 64.5°, suggesting the successful preparation of silver nanoparticles by L-AA. As for the PANI/rGO/Ag/FP composite, the sharp dominant peak at 25.1° was attributed to the reflection of PANI particles. After the formation of PANI, the specified peaks of silver nanoparticles gradually became indistinct, the reason of which was that the fresh layer of PANI covered on the surface of rGO/Ag/FP film had weakened the XRD characteristics of them. Remarkably, the XRD pattern of the PANI/rGO/Ag/FP exhibited a peak similar to that of rGO/Ag/FP hybrid. However, the central point of the peak moved forward and the span of it was widened obviously, demonstrating the existence of PANI. In conjunction with XRD, Raman spectra of the rGO/Ag/FP and PANI/rGO/Ag/FP hybrids were displayed in Figure 2b to make a comprehensive analysis of the chemical bonding structure. As expected, typical Raman signals corresponding to rGO were found from the two-target spectrum. Shown in Figure 2b, prominent peaks located around 1350 cm^−1^ and 1590 cm^−1^ were attributed to the D band and G band, respectively. It was worth noting that the Raman spectra of PANI/rGO/Ag/FP composite also displayed two new peaks at 1165 cm^−1^ and 1503 cm^−1^, which suggested the formation of PANI on the surface of rGO/Ag/FP film [36,37]. Moreover, the low intensity peak ranging from 600 cm^−1^ to 800 cm^−1^ was also assigned to the vibrations of PANI.

**Figure 3:**
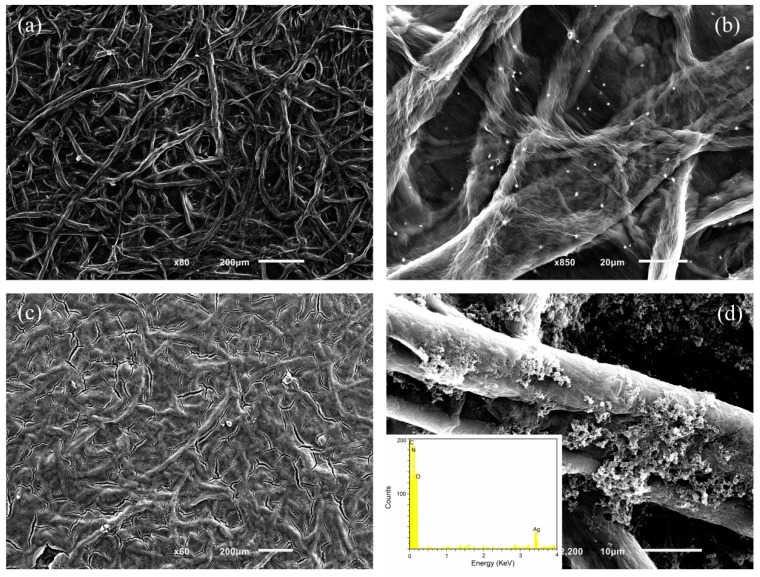
(**a**,**b**) SEM images of the rGO/Ag/FP composite; (**c**,**d**) SEM images of the PANI/rGO/Ag/FP composite.

The structure and morphologies of the rGO/Ag/FP and PANI/rGO/Ag/FP composite materials were further explored by SEM and TEM, respectively. As shown in Figure 3a, the star-like structure of the silver particles were evenly decorated on the surface of rGO sheet with cellulose fibers as the skeleton. It can be seen clearly that each fiber of the FP was encapsulated with rGO/Ag hybrid, forming a three-dimensional electrochemical active structure, which was beneficial to produce more available specific surface area. On one hand, this suggests that the three-dimensional and porous structure possessed large electrochemical active surface area and facilitated the fast diffusion of ions in electrolyte, which come about the enhancement of capacitive property of electrode. On the other hand, the fully homogeneous wrapped rGO/Ag hybrid prepared by the reduction reaction can effectively increase the electrical conductivities and electrochemical performances of the composite electrode compared with the pure FP. The zoomed image of rGO/Ag/FP is presented in Figure 3b. This high power SEM view shows that a wrinkled layer of rGO provided a dramatic backdrop to the small but brightly silver particles display on the surface of rGO/Ag/FP hybrid. As shown in Figure 3c, after chemical polymerization, a dense mass of PANI combined well with the rGO/Ag/FP hybrid, forming a whole tightly. This indicated that the porosity of FP plays a very good role to be filled with activities, which could obtain higher electrochemical performances as well as larger specific surface area. When focusing on the cellulose fiber of the PANI/rGO/Ag/FP hybrid, it can be seen from Figure 3d that agglomerates of granular appearance of PANI located near the surface of rGO/Ag encapsulated FP fibers or filled the pores of the composite. Considering the mechanism of the synergistic effect of PANI/rGO/Ag/FP hybrid, the mechanism of the synergistic effect of this combination technology indicated the unique and outstanding electrochemical properties of the as-prepared electrode. Furthermore, the related Energy dispersive X-ray Spectroscopy (EDS) spectrum (Figure 3d inset) indicated the C and N signal from PANI as expected. The C, O and Ag signals came from the rGO and silver particles, respectively.

**Figure 4:**
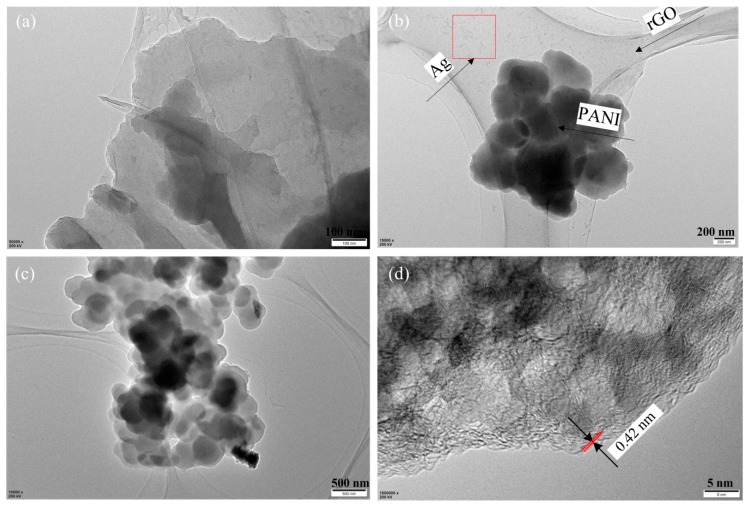
TEM images of PANI/rGO/Ag/FP electrode. (**a**) TEM image of rGO; (**b**) TEM image of PANI/rGO/Ag; (**c**) TEM image of PANI/rGO; (**d**) TEM image of rGO.

To clearly observe the morphology of the as-synthesized electrode material, the TEM images of PANI/rGO/Ag/FP hybrid are displayed in Figure 4. As revealed in Figure 4a, the transparent and wrinkled rGO prepared by the reduction method exhibited a typical two-dimensional paper-like structure with different number of layers stacking each other, which represented the intrinsic morphology of graphene. Moreover, Figure 4b gives a clear image that many black blocks and spots were significantly observed on the layered rGO sheets, indicating the morphology of PANI agglomerates and uniform dispersed silver nanoparticles, respectively. It can be seen in Figure 4c that the agglomerated PANI nanoparticles were in a spheroidal distribution with the diameter of 200~300 nm. A high-resolution TEM of rGO image in Figure 4d showed that the measured layer spacing of rGO was 0.42 nm in the edge of the hybrid, which was in accordance with other reported values [38,39,40].

### 3.2. Electrochemical Performances

**Figure 5:**
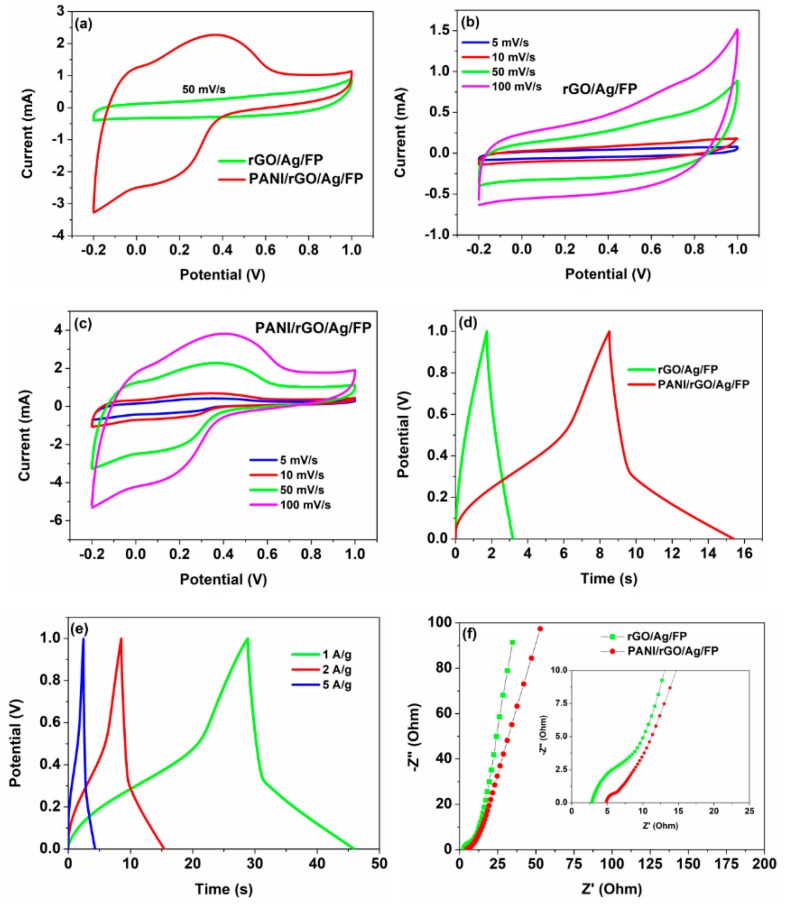
(**a**) Cyclic voltammetry (CV) curves of the rGO/Ag/FP and PANI/rGO/Ag/FP electrodes under the scanning rate of 50 mV/s; (**b**,**c**) CV curves of the rGO/Ag/FP and PANI/rGO/Ag/FP electrodes under different scanning rates, respectively; (**d**) galvanostatic charge-discharge (GCD) curves of rGO/Ag/FP and PANI/rGO/Ag/FP electrodes at the current density of 2 A/g; (**e**) GCD curves of PANI/rGO/Ag/FP electrode at different current densities; (**f**) Nyquist plots of rGO/Ag/FP and PANI/rGO/Ag/FP electrodes.

The electrochemical performances of the as-fabricated PANI/rGO/Ag/FP hybrids were studied systematically by CV, GCD and EIS methods, respectively. For comparative analysis, the capacitive properties of the rGO/Ag/FP composites were also investigated under the same testing conditions. The CV curves of PANI/rGO/Ag/FP and rGO/Ag/FP hybrids at the same scanning rate of 50 mV/s are shown in Figure 5a, respectively. By contrast, the CV curves of PANI/rGO/Ag/FP and rGO/Ag/FP composites differed greatly. Apparently, the CV curve of rGO/Ag/FP displayed a near rectangle in shape and no redox peaks of rGO/Ag/FP hybrid were observed, implying a typical double layer capacitance. However, that of PANI/rGO/Ag/FP behaved a dramatic pseudocapacitance with a couple of sensitive and reversible redox peaks, which were assigned to leucoemeraldine-EB (A1/C1) and EB-pernigraniline (A2/C2) transformation of PANI [41]. Besides, the absolute area of the CV curve of PANI/rGO/Ag/FP composite was significantly greater than that of rGO/Ag/FP, demonstrating a higher specific capacitance of PANI/rGO/Ag/FP. The first reason for this can be put down to the high pseudo-capacitance energy storage characteristic and good conductivity of PANI formed on the surface of rGO/Ag/FP composite. The other reason was that the hybrid electrode of PANI/rGO/Ag/FP results in a synergistic effect happened among PANI, rGO and Ag particles. Figure 5b,c display the CV curves of rGO/Ag/FP and PANI/rGO/Ag/FP under different scanning rates of 5 mV/s, 10 mV/s 50 mV/s and 100 mV/s, respectively. It was obvious that few changes have been observed on the CV curves of rGO/Ag/FP and PANI/rGO/Ag/FP hybrid with the scanning rates increasing, which suggested the rate performance of PANI/rGO/Ag/FP was as good as the almost ideal supercapacitor of rGO/Ag/FP composite. It was worth noting that the oxidation peaks of the PANI/rGO/Ag/FP hybrid shifted towards a positive direction, but the reduction peaks moved negatively as the scanning speed increased. The redox peaks were still clearly visible even at the high scanning rate of 100 mV/s, indicating the good rate ability discussed above. In order to contrast and analyze, the GCD curves of rGO/Ag/FP and PANI/rGO/Ag/FP hybrids were plotted on the same graph with the current density of 2 A/g. As shown in Figure 5d, the GCD curve of rGO/Ag/FP displayed symmetrical triangular waves, demonstrating the excellent electrochemical reversibility. However, after preparation of PANI, the shape of the GCD curve of the PANI/rGO/Ag/FP film deformed measurably compared to that of rGO/Ag/FP. It suggested the pseudocapacitance characteristics of PANI were exerted adequately, resulting in the hybrid capacitance of PANI/rGO/Ag/FP electrode. In addition, the charge and discharge time of PANI/rGO/Ag/FP electrode was significantly reduced compared to the other PANI/rGO hybrid materials. In fact, the uniform distribution of silver nanoparticles on the surface of rGO/FP played an important role in the improvement of electrochemical properties. To further investigate the charge-discharge performances, the results of the study on PANI/rGO/Ag/FP electrode at various current densities of 1 A/g, 2 A/g and 5 A/g are shown in Figure 5e, respectively. It was noticeable that as the current density increased, the charge and discharge time of PANI/rGO/Ag/FP hybrid was greatly shortened. Thus, according to the power density calculation formula above, it was concluded that the faster the current density was, the higher power density obtained. Nyquist plots of the rGO/Ag/FP and PANI/rGO/Ag/FP electrodes from EIS across a wide frequency range of 0.01 Hz to 100 kHz are shown in Figure 5f. Typically, the Nyquist plot consisted of an approximate straight line at low frequencies and one arc curve in high frequencies, representing the ion diffusion resistance and charge transport resistance (R_ct_), respectively. The greater the slope angle of the straight line was, the smaller ion diffusion resistance obtained. Besides, the charge transport resistance can be concluded from calculating the capacitance arc diameter. The horizontal intercept on the Z’-axis in high frequencies was the equivalent series resistance (R_s_) of the electrode, which related to the intrinsic resistance of chemical activities of the electrode, contact resistance at the interface between the electrode and electrolyte and the resistance of electrolyte in total. Apparently, because of the larger surface area and better adsorption performance of rGO, the ion transport rate of rGO/Ag/FP was a little faster than that of PANI/rGO/Ag/FP. However, the smaller diameter of the arc curve in Figure 5f inset demonstrated the low charge transport resistance of PANI/rGO/Ag/FP due to the good conductivity of PANI coated rGO doped by silver nanoparticles composite. Moreover, the X-intercept of Nyquist plots suggested the equivalent series resistance of rGO/Ag/FP was a slightly lower compared with that of PANI/rGO/Ag/FP, which was ascribed to the increase of the intrinsic resistance of PANI/rGO/Ag/FP hybrid after the formation of PANI.

**Figure 6:**
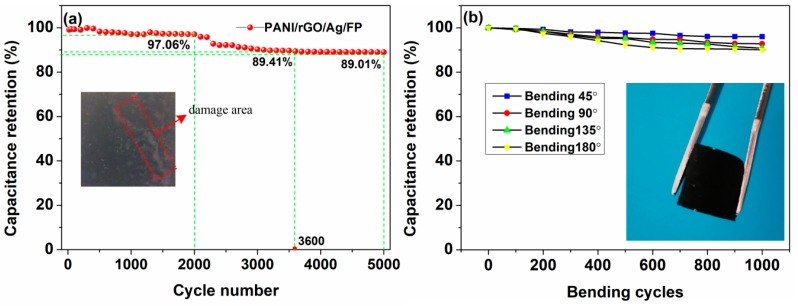
(**a**) The specific capacitance retention rate of the PANI/rGO/Ag/FP electrode over 5000 cycles. (**b**) The specific capacitance retention rate of the PANI/rGO/Ag/FP electrode under different bending degrees.

The long-term cycling tests of PANI/rGO/Ag/FP hybrid were conducted by CV at the scan rate of 50 mV/s for 5000 times. As displayed in Figure 6a, initially, the specific capacitance maintained a high level with 97.06% retention over the first 2000 cycles. However, there was a slight reduction of the specific capacitance when the cycling number approached 3600, which was attributed to the loss of detached active materials falling from the electrode and then travelling to the electrolyte during the charge-discharge process. The image of the sample after cycling tests was displayed in the Figure 6a inset. Finally, the specific capacitance remained in a stable state with the low deterioration rate of 10.99%, indicating an excellent cycle stability of the PANI/rGO/Ag/FP composite. For flexibility, the specific capacitance retention rates of the electrode were also calculated under different bending degrees of (45°, 90°, 135°, 180°) and repeated bending times (1000), respectively. As shown in Figure 6b, although the PANI/rGO/Ag/FP electrode experienced a maximum bending degree of 180° and the highest number of bending times, the calculated retention rate remained as high as 90.1%.

## 4. Conclusions

In summary, a facile silver doped graphene strategy combined with polyaniline was employed to fabricate a lightweight and flexible filter paper based supercapacitor electrode with high electrochemical performance. Due to the large specific surface area of the chemical activities and porous structure of the filter paper, the as-prepared PANI/rGO/Ag/FP hybrid obtained a high surface area and favorable access for ions diffusion in the electrolyte, leading to an enhanced specific capacitance of 437.3 F/g, high energy and power densities of 1133.5 W h/kg and 88.8 kW/kg, respectively. Moreover, the PANI/rGO/Ag/FP also demonstrated an excellent cycling stability, which maintained 89.01% of the initial specific capacitance value after 5000 cycles. The mechanical bending tests of the electrode indicated an outstanding flexibility, which may suffer from excessive and repeated bending deformation. From the above results, the excellent electrochemical performances of the hybrid PANI/rGO/Ag/FP electrode make it a potential candidate for the flexible supercapacitor. Besides, the simple and reliable preparation process of this electrode can be used extensively in the field of energy storage. In addition, the facile method and porous structure of the as-prepared electrode presents the possibility for fabricating high-performance, low cost and environmentally friendly biomass-derivative products with great application potential.
